# Follow, Flex, and Flout: A Relational Frame Theory Account of Flexibility in the Context of Rule-Governed Behavior

**DOI:** 10.3390/bs15060795

**Published:** 2025-06-10

**Authors:** Alison Stapleton, Conor McCloskey, Louise McHugh

**Affiliations:** School of Psychology, University College Dublin, D04 V1W8 Dublin, Ireland; dralisonstapleton@gmail.com (A.S.);

**Keywords:** pliance, tracking, health, flexibility, rule-governed, relational frame theory

## Abstract

Being able to change what we are doing when a behavior no longer serves us is important for our health and wellbeing. In the context of rule-governed behavior, changing one’s behavior in line with shifting contingencies is often described as being “flexible”, with many basic laboratory experiments operationalizing flexibility as deviations from a given rule that no longer results in reinforcement. And yet flexibility is not just about flouting rules; sometimes, being flexible means persisting. This paper unpacks flexibility in the context of rule-governed behavior from a relational frame theory perspective, outlining applied examples relevant to health behaviors.

## 1. Introduction

Engaging in behaviors that support our health and wellbeing involves following simple rules—for example, “eat balanced meals”, “exercise regularly”, “avoid smoking”, and more. However, the way we follow these rules can make all the difference. Imagine a daily gym-goer who, motivated by social media likes, rigidly follows their workout routine because, for them, a missed session runs the risk of public disapproval. Their adherence, while seemingly beneficial, could inadvertently lead to burnout or injury. That said, outright flouting of such rules in favor of immediate rewards (e.g., smoking) can be equally problematic for long-term health. The capacity for flexible rule-following, that is, adjusting one’s actions in line with real-world contingencies, may be far more valuable than inflexible obedience to rules. The purpose of the present perspective paper is to illustrate how rule-governance relates to the acceptance and commitment therapy (ACT) concept of psychological flexibility in the novel context of health (Narula, 2024). Utilizing relational frame theory (RFT) as a framework, we describe the types of rule-following per RFT and outline how they may be relevant to health and wellbeing, concluding with a novel case example.

## 2. Defining Rule-Governance

In seeking to understand the role that rule-following plays in health behaviors, a basic analysis of rule-following is needed. In its traditional behavioral account, rule-governed behavior refers to behaviors that are influenced not through direct contact with contingencies of reinforcement but instead through verbal stimuli that specify the effects of contingencies. In comparison with learned behavior, rule-governed behavior is typically acquired more quickly and generally understood to be more suited to situations where controlling contingencies is remote or complex (Skinner, 1966; Vaughan, 1989). This distinction is particularly relevant in the context of health and wellbeing. For example, consider smoking cessation. The consequences supporting smoking cessation may be drowned out by smoking, itself a reinforcer (Cohen & George, 2013). The consequences of smoking cessation would likely be far too remote for direct contingencies alone to maintain due to the short-term appetitive nature of smoking, in contrast to its probabilistic aversive long-term consequences.

RFT provides one thorough analysis of rule-governance (Hayes et al., 2001). In its simplest form, RFT is a bottom-up account of how human behaviors, including language use, interact with wider environments, with a particular focus on the role that relating stimuli to one another plays in this (Stewart & Roche, 2013). RFT allows for a functional understanding of rule-governance by providing a framework and terminology for analyzing rule-following and the functions that are transformed by rules (O’Hora & Barnes-Holmes, 2004). In the original RFT account of rule-governance, rules were understood to operate as specific and distinct relational networks that were controlled by different underlying contingencies of reinforcement, with these controlling networks being presented as distinct functional classes of rule-following (Hayes et al., 2001).

Transformation of stimulus function explains how arbitrary stimuli, such as the words stated in a rule, specify and acquire the properties of other stimuli, such as the concepts denoted by the words stated in a rule (O’Hora & Barnes-Holmes, 2004). In this way, transformation of function provides rules with behavior-controlling properties. As such, from an RFT perspective, rules are generally understood as being complex relational networks that include frames of coordination (i.e., for specification) and conditional (e.g., “if… then”) or temporal (e.g., “before… after”) frames that transform the functions of framed events (O’Hora & Barnes-Holmes, 2004). Similarly, rule-governed behavior is generally understood as behavior under the control of two sets of contingencies, one a direct contingency and the other involving a verbal antecedent (Zettle & Hayes, 1982).

To illustrate this, imagine working with a personal trainer to achieve your fitness goals. If the trainer were to say something like “brisk daily walks will help you to meet your goals”, you may understand this as “if I walk each day, then I will achieve my goals”. Consequently, because you want this outcome, you walk each day. So, in this example, you understand and follow a rule delivered by the trainer. Applying a relational frame interpretation, we might say that you have coordinated word classes (e.g., “walk”) with some event classes (e.g., the actual act of walking), further relating these verbal classes conditionally, with “if” and “then” bringing additional arbitrary relations to bear. Understanding the rule in this way alters the functions of walking, with transformations of stimulus function connecting the act of “walking” to the desirable consequence of “achieving goals”. As such, when next invited on a walk with colleagues during lunchtime, you may decide to join them, especially if you have not yet walked that day. You now experience your colleagues inviting you on a walk differently from how you experienced it before the rule since before the rule, an invitation from colleagues to walk at lunch may have simply been neutral or even mildly aversive (e.g., interrupting work or causing social discomfort). After the rule is understood and related to personal values, such as those underlying one’s fitness goals, the function of “walking” is transformed. Consequently, an invitation to walk evokes behavior (joining the walk) because it is relationally connected to achieving personally significant outcomes. So now, when colleagues invite you to walk, that verbal stimulus functions as a cue to engage in goal-aligned behavior because it participates in a relational network that includes valued outcomes. This differs from an establishing operation in traditional behavior analysis because establishing operations alter the value of a reinforcer and the probability of the behavior that has historically contacted that reinforcer.

Per Zettle and Hayes (1982), a complete analysis of the rules provided by other people must incorporate functional units of speaker’ behavior, formal aspects of speech, and functional units of listener behavior. In terms of listener behavior, Zettle and Hayes (1982) proposed three main functional units, specifically pliance, tracking, and augmenting, each of which seems relevant to psychological flexibility.

### 2.1. Pliance

Pliance refers to rule-following controlled by a history of arbitrary speaker-mediated reinforcement of the correspondence between behavior and rules (Hayes et al., 1998; Ruiz et al., 2019). In simpler terms, pliance means rule-following for the sake of rule-following itself. If a social community fails to appropriately contextualize pliance for an individual, then pliance will likely become generalized, dominating their behavioral repertoire (Ruiz et al., 2019). Topographically, generalized pliance might look like “excessive people-pleasing” or “hypersensitivity to peer pressure” because people displaying generalized pliance are attuned to apparent arbitrary social approval (Törneke et al., 2008). Breaking this concept down further, pliance means doing as you are told (i.e., complying) purely because “compliance” has paid off in the past; thus, you expect a desirable outcome when you comply with someone’s rule (i.e., there is an apparent arbitrary socially mediated consequence available).

With pliance, “arbitrary” means that the consequences depend on the detection of rule-behavior coordination that alters the behavior of the verbal community, who then consequate the rule-following. As Hayes et al. (1989, p. 204) argues, “with pliance, the consequences must be socially mediated because only a social/verbal community can discern the presence of a rule and check for behavior that corresponds with it. The fact of social mediation, however, is background; the foreground issue is that the socially mediated consequences are for rule-following”.

Pliance in and of itself is not problematic; sometimes, we want people to learn from our advice rather than their direct experience. However, according to Ruiz et al. (2019), because pliance is the first type of rule-following to develop, it typically generalizes during childhood, meaning arbitrary social approval becomes a child’s main source of reinforcement. Research suggests that generalized pliance decreases with age (Salazar et al., 2018), likely due to interactions contextualizing pliance and shaping tracking. However, in the absence of such interactions, pliance will likely remain generalized (Ruiz et al., 2019). Generalized pliance is conceptualized as highly problematic, likely complicating the development of personal values and potentially precipitating ineffective situational sensitivity (O’Connor et al., 2019; Ruiz et al., 2019). In addition, because social contexts typically promote engaging in experiential avoidance in response to aversive private events, people displaying generalized pliance may be more likely to behave in psychologically inflexible ways (Ruiz et al., 2019).

Applied to health, imagine your doctor advises you to reduce your sugar intake. Immediately after your doctor visit, you may pliantly adhere to this “reduced sugar” rule. However, it is difficult to maintain the conditions controlling pliance for prolonged periods. For example, perhaps your next doctor visit is some months away, and they do not monitor your intake in the interim. The consequences of consuming sugar may be more reinforcing than the distal ones from your doctor, so if they are all that controls your reduced sugar intake, you may abandon the rule soon after your visit. Pliance requires an “ever-present rule giver” (Zettle & Hayes, 1982, p. 84) or an ever-present monitor, so your doctor could incorporate wearable health technology into your care plan (e.g., by asking you to wear a device that measures your blood sugar levels). However, contacting the consequences supporting pliant rule-following will usually be more probabilistic than contacting the consequences involved in direct contingencies. For example, your device could be faulty, or your doctor could be overstretched and unable to consequate your adherence between visits. For this reason, reliance on pliance seems unwise long-term. Ultimately, attending to the natural consequences of our behavior seems like a relatively more stable path towards the long-term maintenance of health behaviors since those depend entirely on the form of our behavior, not social detection, and are thus more certain.

### 2.2. Tracking

Tracking refers to rule-following controlled by an apparent correspondence between a rule and the way the environment is arranged, independent of the delivery of the rule (Barnes-Holmes et al., 2001; Hayes et al., 1998). With tracking, consequences are natural and non-arbitrary (Hayes et al., 1998; Ruiz et al., 2019). This means it is the precise form, frequency, and/or situational sensitivity of the listener’s behavior in a given context (i.e., not social detection that rule-following has occurred) that allows the listener to contact the consequences of following the rule. While the consequences supporting tracking may be social (i.e., when a rule is followed to contact natural non-arbitrary social consequences, such as sexual arousal which is determined, to a degree, by the form of sexual stimulation; Waldeck et al., 2019), differing from pliance, the consequences do not need to be social or speaker-mediated: “the consequences of an action are determined completely by the topography of the action itself in a given situation” (Hayes et al., 1989, p. 204).

Returning to the previous example of your doctor advising you to reduce your sugar intake, tracking might look like adhering to the rule because doing so allows you to avoid crashing energy levels. In the context of reducing one’s sugar intake, given the initial risk of sugar withdrawal, the benefits of pliance are clear. Appetitive consequences like stable energy levels may be too remote to promote adherence. Therefore, as a more effective way to encourage a reduced sugar intake, tracking may be utilized to shape sensitivity to these remote consequences (e.g., your doctor asking you to note your food intake and energy levels throughout the day).

By definition, relative to pliance, tracking is more attuned to shifting environmental contingencies (i.e., more conducive to flexible, context-sensitive rule-following). However, tracking may also become problematic. Villatte et al. (2016) detail problematic types of tracking, namely inapplicable tracking and inaccurate tracking, in addition to a subtype of inaccurate tracking where tracking leads to “adaptive peaks”.

Briefly, inapplicable tracks are defined as “accurate” (i.e., accurately specifying the natural consequence), impossible rules (e.g., rumination, excessive planning around uncontrollability, etc.). Often, inapplicable tracks take the form of rules we have for other people (i.e., inapplicable tracks fail to say anything about what we can do since they typically specify favorable outcomes based on others’ behavior). In other words, our inapplicable tracks do not *apply* to us as a rule-follower. In the context of sugar intake, an inapplicable track might look like “if my partner didn’t buy sugary snacks, I wouldn’t eat as much sugar”, which is accurate in that such a circumstance may actually lead to a reduced sugar intake but is not in the realm of control of the person creating the track. Of course, inapplicable tracks in and of themselves are not problematic; rather, issues stem from the ways in which we respond to inapplicable tracks. Are we fighting with our partner or discussing ways we can both meet our needs and support each other’s health goals?

Inaccurate tracks are defined as rules that specify a functional relationship that does not align with actual broader experience. Often, inaccurate tracks are vague and absolute (overgeneralized, e.g., “I always get hurt when…”) and fail to simultaneously account for short- and long-term consequences (i.e., prioritizing short-term gain regardless of consequential long-term pain). An example of an inaccurate track is continuing to smoke cigarettes rather than quitting due to the perceived increases in stress associated with the initial phase of smoking cessation; “if I quit smoking, I will feel stressed”. As another example, in the context of nutrition, an inaccurate track might be “a nutritious diet does not include fat”. Given that fat is an essential part of a balanced diet that helps us to absorb fat-soluble vitamins (and more), this rule is inaccurate.

A subtype of inaccurate tracks, tracking leading to “adaptive peaks”, refers to rules that specify a desirable consequence that overwhelms other desirable consequences (i.e., we are receiving positive reinforcement but not responding optimally). Often, with this subtype, interventionists will attempt to orient clients toward the sources of reinforcement that are currently being missed. One example of tracking leading to an adaptive peak is drinking excessive amounts of protein shakes when trying to gain muscle despite experiencing digestive issues as a result. In this example, although there are other ways to meet our protein goals, avoid lean muscle mass loss, and avoid digestive issues, the short-term pay-off from consuming excessive protein shakes may prevent us from exploring these other avenues. The apparent successful short-term workability overwhelms the potential gains from other sources of reinforcement.

Having described pliance and tracking, it seems important to discuss whether they are functionally distinct. Kissi et al.’s (2017) systematic review of pliance, tracking, and augmenting concludes that “it is difficult to determine the extent to which the concepts of pliance, tracking, and augmenting allow for relatively precise experimental analyses of distinct functional classes of behavior” (p. 695). Indeed, there is limited research on pliance, tracking, and augmenting. However, Kissi et al. (2017) argue in favor of using pliance and tracking as middle-level terms, emphasizing an ultimate need to relate these middle-level terms to basic laboratory studies (i.e., they do not recommend abandoning these terms). In addition, as defined, there is a meaningful difference between plys (pliance-based rules) and tracks (tracking-based rules), specifically in the probability that consequences will be contacted, which seems highly clinically relevant. Compared to behavior controlled by arbitrary speaker-mediated consequences, behavior maintained by natural consequences is more likely to be reinforced, perhaps promoting more expansive repertoires. Ultimately, further research is needed to identify whether pliance and tracking are distinct classes and whether it is clinically useful to conceptualize rule-following per plys and tracks.

### 2.3. Augmenting

Augmenting (involving rules termed “augmentals”) is often described as a distinct unit of rule-governed behavior. However, the way augmenting functions is by altering the reinforcing or punishing features/functions of the specified consequences of plys/tracks (i.e., it works in conjunction with pliance and tracking). There are two types of augmentals discussed within the literature, namely formative and motivative augmentals (Barnes-Holmes et al., 2001; Hayes et al., 1998). Formative augmentals establish new consequences for previously neutral stimuli (Hayes & Wilson, 1993). Motivative augmentals change the reinforcing value of consequences specified in the rule, temporarily altering the degree to which established consequences serve as reinforcers or punishers (Ju & Hayes, 2008; Törneke et al., 2008). Simply put, motivative augmentals alter our interest in existing consequences (Villatte et al., 2016). This is often achieved by bringing distant consequences to the present via language or encouraging clients to contact natural and social reinforcers (Barnes-Holmes et al., 2001; Ju & Hayes, 2008; Villatte et al., 2016). Motivating operations, from traditional behavior analyses, change the effectiveness of a reinforcer or punisher by altering environmental or biological conditions (e.g., hunger increases the value of food). In contrast, motivative augmentals, from the RFT perspective, change the perceived value of consequences through language and relational networks. They influence behavior by altering the meaning or significance of outcomes without changing the physical environment. While motivating operations impact contingency-shaped behavior directly, motivative augmentals influence rule-governed behavior. Notably, some researchers approach augmentals as verbal motivating operations. However, there is no consensus on the appropriateness and usefulness of this approach.

In health contexts, motivative augmentals often appear in motivating statements such as “being active helps me feel like myself again” or “preparing balanced meals is how I take care of the people I love”. These statements do not simply describe contingencies, as in tracking, or reflect arbitrary speaker-mediated control, as in pliance. Instead, they involve the transformation of the function of the consequences by connecting them to personally meaningful outcomes. In a health context, formative augmentals may take the form of the nutritional information associated with specific food. Augmentals sometimes strengthen the influence of plys and tracks and at other times weaken them. For example, a person may begin exercising to meet social expectations, but over time, as they contact the benefits of more movement, a new augmental rule may emerge that links physical activity to their sense of purpose. Understanding how augmentals operate may offer ways to develop psychological flexibility in health interventions, not by eliminating rules but by reshaping their functions to connect them with what matters to the individual, that is, an individual’s freely chosen values.

Thus far, we have applied Zettle and Hayes’s (1982) and Villatte et al.’s (2016) RFT-based consistent accounts of rule-governance to the domain of health. Before progressing, it is important to note that other optional add-ons to RFT have conceptualized rule-governance differently. For example, a researcher using the hyperdimensional multilevel (HDML) framework might instead classify rule-following across levels of relating according to whether it is high or low in coherence, complexity, derivation, and flexibility (Harte et al., 2020). Per this approach, it may be difficult to distinguish between plys and tracks, as perhaps they would only differ somewhat consistently in terms of observed flexibility. Nonetheless, the HDML may prove to be a valuable tool for analyzing rule-following. For example, it seems clinically relevant to identify whether relations are newly derived or metaphorical “well-worn” paths of relating. Ultimately, a blend of differing approaches may equip professionals best to conceptualize and support context-sensitive rule-following. Indeed, as proponents of the HDML note, “the dimensions and levels specified in the HDML framework may be helpful in refining [pliance, tracking, and augmenting] in a functional-analytic way” (Harte et al., 2020, p. 380).

## 3. What Constitutes Inflexible Rule-Following?

Rigid rule-following refers to instances where someone persists in following a rule despite contacting aversive consequences and/or failing to contact appetitive consequences (i.e., a problematic rule is an unworkable rule). In theory, problematic rule-following is associated with insufficient or inappropriate contextual control, a “drowning-out” of contingencies, and/or insufficient or inappropriate generalization. Relatedly, in theory, attending to only what is understood in the rule is likely to limit our experience; we have fewer response options available to us when we do not respond to stimuli that are not specified/not understood in the rule. Put more simply, when we fail to attend to the net consequences of our behavior, we can become stuck in unhelpful loops. Rather than optimizing our behavior, rules of this type may be viewed as self-limiting and problematic. In theory, problematic rule-following is not limited to one presentation or neurotype; it is argued to be a universal potentiality among all verbally able (in the RFT sense) people. For example, rules relating to self-advocacy (“I don’t deserve respect”), emotional expression (“I shouldn’t have these feelings”), and productivity (“I’m not working hard enough”) may be present in clinical and non-clinical populations (i.e., may show up regardless of one’s neurotype). So, anyone can display rigid rule-following.

Regarding psychological flexibility (per the hexaflex; see Levin et al., 2012; McCracken & Morley, 2014) and rule-governance, acceptance may look like “sitting with” or allowing unwanted and uncomfortable internal experiences that show up when rules we hold conflict with our values or compete with other desirable contingencies. Defusion might look like broadening stimulus control, deliteralizing the content of self rules (i.e., coming to recognize them as a mere collection of words), and recognizing that the self is distinct from the rule (e.g., naming the rule when we notice it showing up). Somewhat overlapping with defusion, self-as-context may look like situating the speaker as both distinct from and the container of their rules, while discovering a safe, stable place from which to view self-content. Awareness of the present moment may facilitate more accurate tracking (i.e., with greater attention paid to our immediate environment). Values may emphasize the importance of not allowing our behavior to be solely controlled by consequences from others while signposting other viable sources of reinforcement. Committed action may look like doing what matters to us, regardless of our unwanted and uncomfortable internal experiences (so long as that is safe and not overstretching us). Committed action may also look like persistence in novel discovery and tracking behaviors in the absence of our former reinforcers.

In terms of rigid rule-governance, research has identified negative correlations between generalized pliance and psychological flexibility (Waldeck et al., 2019), in addition to positive correlations between tracking and psychological flexibility (Ruiz et al., 2020a). Although few empirical studies have directly examined the effects of ACT on pliant rule-following, they found for repetitive negative thinking that ACT was associated with lesser problematic pliance post-intervention (Ruiz et al., 2020b; Salazar et al., 2020). Notably, to date, no studies have reportedly examined the effects of ACT on tracking.

## 4. A Case Example

This section illustrates how the same verbal rule can support psychological flexibility or inflexibility depending on its controlling contingencies. The example is designed to demonstrate the interplay of pliance, tracking, and augmenting in a real-world health context, highlighting how apparent behavior changes may mask inflexible rule-following unless the function of the rule is examined.

Let us take the domain of food. Even before you finish that sentence, rules are likely crowding in. Maybe you are noticing thoughts like “there’s good food and bad food”, “eat less, exercise more”, “all food fits”, “but fat is unhealthy”, and so on. Health domains like diet, exercise, and substance use are saturated with socially transmitted rules, some widely endorsed, some contested, and many shaped by familial, cultural, and digital contingencies. These are ideal contexts for exploring rule-governed behavior because the contingencies are rich, the histories are long, and the social reinforcers potent. Importantly, they are also domains where rigid rule-following or indiscriminate flouting can both lead to harm.

Take the rule “eat clean”. When this is followed flexibly, it might guide someone toward balanced meals that support their long-term health goals. However, when this same rule is maintained by generalized pliance, driven by the need for social approval or fear of judgment, it can contribute to harmful dietary restriction or obsessive eating practices. On the other hand, rigidly rejecting all food-related rules (e.g., “all food rules are harmful”) without sensitivity to tracking real outcomes, such as medical dietary needs, may also produce unhelpful consequences. What these examples illustrate is that the problem is not the rule itself but the functional relationship that the individual has with the rule.

Consider Emma, a 28-year-old woman recovering from an eating disorder. She initially attributes her restrictive eating patterns to a desire to be “healthy”, a rule she picked up from fitness influencers that was reinforced by online praise. Despite physical signs of malnourishment and social withdrawal, Emma continues to follow strict food rules because she fears disapproval if she deviates. Her adherence is driven primarily by pliance, maintained by arbitrary speaker-mediated consequences.

In therapy, Emma is encouraged to “listen to her body”. On the surface, this appears to be a shift away from rigid rules toward more flexible, body-based tracking. However, Emma understands this invitation as a new rule to be executed precisely. She begins documenting her hunger levels and meal satisfaction scores with the same rigidity as before. The surface form of her behavior has changed, but its function, compliance to perceived external standards, has not. This is an example of pliant substitution, where the function of rule-governed behavior remains unchanged despite a new verbal topography.

It is only when Emma and her therapist explore why she values health and how her current behaviors do or do not serve those values that true change begins to occur. They work together to identify moments where she can choose actions based on her own values (e.g., sharing an unplanned meal with friends) rather than on socially prescribed ideals. She learns to identify situations where she can choose a different action, not because it is the “right” rule but because it better aligned with what matters to her in that moment. This transition reflects a shift toward augmenting, and new rules emerge that connect behavior with intrinsic, meaningful outcomes rather than arbitrary social consequences.

For example, the rule “sharing meals helps me feel connected to people I care about” functions differently from “I must eat clean to be good”. It is not simply another ply to follow but an augmental that recontextualizes Emma’s behavior in terms of values. From an RFT perspective, this transformation alters the reinforcing function of the consequences and supports psychological flexibility. This case also helps illustrate the concept of inapplicable tracking. Consider the rule “if my partner didn’t buy sugary snacks, I wouldn’t eat as much sugar”. This statement is accurate in identifying a cue in the environment (i.e., the presence of snacks), but it is impossible in that it specifies a behavior contingency dependent on someone else’s actions, something the individual cannot directly control. Without intervention, such rules may foster passivity, resentment, or avoidance rather than value-based behavior.

To summarize the key concepts Emma’s case exemplifies, first, we can see generalized pliance, rule-following maintained by the need to avoid shame or gain approval. Then, we can see pliant substitution, where there is a superficial rule change without functional change. We then saw tracking misapplied, with rigid adherence to the rule “listen to your body”, and augmenting, where there is a shift toward value-based rules that recontextualize consequences and promote flexibility. Finally, we saw an example of inapplicable tracking, where a rule accurately describes contingencies but is impossible to act on due to its dependence on another’s behavior.

This example illustrates that psychological flexibility in the context of rule-following depends less on the content of rules and more on their function. Encouraging clients to adopt new health-related rules may backfire if these rules are simply adhered to pliantly. A functional analysis, not topographical change, is what determines meaningful behavior change. The interpretation offered here aligns with empirical and conceptual analyses of rule-governed behavior in RFT (e.g., Hayes et al., 2001; Ruiz et al., 2019) and ACT’s focus on psychological flexibility (Levin et al., 2012). Emma’s case is not intended as generalizable clinical data but as a conceptually coherent narrative that illustrates the concepts presented in this paper. By embedding consistent RFT constructs (pliance, tracking, and augmenting) into a single applied context, we aim to demonstrate their relevance to health and intervention design.

## 5. Conclusions

In health behavior research and intervention design, then, it is crucial to clarify how rule-following is operationalized and what kinds of contingencies are at play. Is the person persisting or adapting in ways that are sensitive to shifting contexts? Are we reinforcing behaviors that serve long-term values or simply maintaining social compliance? Without this clarity, we risk designing interventions that look successful in the short term but fail to promote genuine psychological flexibility. Health is a domain full of rules, and behavior change is often the goal. If we want to support flexible, sustainable change, we need better ways of identifying when rules are helping or hindering and more functional analytic precision in our interventions.

RFT offers an advantageous position for understanding flexibility in the context of health-related behavior change through allowing a shift in focus from the form of rules to their function, helping to avoid common issues in intervention design. RFT can facilitate distinctions between classes of rule-following, each associated with specific sources of control, and additionally integrates well with applied models such as ACT, offering testable predictions about processes of change.

However, there are limitations to the extant RFT accounts. Empirically, the distinctions between pliance, tracking, and augmenting are still underdeveloped, with a limited number of studies having directly tested the function distinction between these categories in real-world settings (Kissi et al., 2017). Most notably, issues in the study of pliance persist with a need for development in how arbitrary and natural consequences are operationalized (McCloskey et al., 2024). In practice, rule-following rarely presents in pure forms, and multiple functions may operate simultaneously or shift dynamically within and across contexts. This functional complexity poses challenges for reliable assessment and intervention. Finally, while the proposed framework is conceptually sound, future research should be conducted to determine the clinical utility of targeting specific rule functions in promoting sustainable health behavior change. This paper has argued that to follow, flex, or flout effectively, we must go beyond form and attend to function, especially when the stakes, as in health, are high.

## Data Availability

No new data were created or analyzed in this study.

## Abbreviations

The following abbreviations are used in this manuscript:

RFT	Relational Frame Theory
ACT	Acceptance and Commitment Therapy
